# Roles of nucleotide metabolism in pancreatic cancer

**DOI:** 10.3389/fimmu.2025.1637768

**Published:** 2025-10-22

**Authors:** Quanlin Liu, Jiahua Liu, Shige Wang, Nabuqi Bao, Xinya Zhao, Lei Wang

**Affiliations:** ^1^ First Affiliated Hospital of Dalian Medical University, Dalian, China; ^2^ Jinqiu Hospital, Shenyang, China

**Keywords:** nucleotide metabolism, pancreatic cancer, therapeutic targets, pathogenesis, metabolic reprogramming, KRAS mutation, immune microenvironment

## Abstract

Nucleotide metabolism plays a pivotal role in the onset and progression of various human diseases, including pancreatic disorders. As fundamental biomolecules, nucleotides are essential for DNA and RNA synthesis, energy production, and cell signaling. Disruptions in nucleotide metabolic pathways have been linked to altered cell proliferation, apoptosis, and immune responses—critical processes in the development of pancreatic diseases. In pancreatic cancer, metabolic changes in nucleotides facilitate rapid tumor cell proliferation and enhance chemotherapy resistance. Recent studies have concentrated on identifying specific enzymes and pathways within nucleotide metabolism as potential therapeutic targets. Targeted interventions, such as modulating RRM2, TS, and other key enzymes or disrupting the PI3K/AKT/mTOR pathway, have demonstrated potential in reducing tumor growth and inflammation in pancreatic tissue. This review provides an overview of the latest advancements in the understanding of nucleotide metabolism in pancreatic cancer pathogenesis, emphasizing diagnostic and therapeutic strategies that may improve patient outcomes.

## 1 Introduction

Abnormalities in nucleotide metabolism have been shown to play a critical role in the onset, progression, and treatment response of acute pancreatitis (AP), chronic pancreatitis (CP), and pancreatic cancer (PC). PC, an aggressively invasive malignancy with an exceptionally high mortality rate, is often referred to as the “King of Cancers.” As one of the deadliest cancers globally, its subtle early symptoms frequently result in diagnosis at advanced stages. Treatment options for PC remain limited, and the prognosis is poor, highlighting the importance of a deeper understanding of its molecular mechanisms to develop more effective therapies (1).

In pancreatic diseases, disruptions in cellular metabolism are fundamental. Nucleotide metabolism, a key aspect of cellular metabolism, serves not only as a critical precursor for DNA and RNA synthesis but also plays an essential role in biological processes such as cell proliferation, survival, aging, and apoptosis. In PC cells, nucleotide metabolic pathways are frequently reprogrammed to support rapid tumor cell proliferation and resistance to chemotherapy (2). Research has clarified that nucleotide metabolism operates primarily through two biosynthetic pathways: the *de novo* synthesis pathway and the salvage pathway (3). PC cells rely heavily on the *de novo* synthesis pathway to meet the demands of their accelerated growth.

Moreover, nucleotide metabolism is regulated by several signaling pathways, including PI3K/Akt, mTOR, and p53, which significantly influence the initiation and progression of PC. Recent studies have demonstrated that targeting nucleotide metabolism can not only impede tumor growth but also enhance chemotherapy sensitivity (4, 5). Ongoing research is actively identifying new nucleotide-related tumor markers in cancer (6, 7), with increasing attention being paid to personalized treatment approaches for patients (8).

Thus, a comprehensive investigation into the role of nucleotide metabolism in PC offers insights into potential mechanisms for its treatment and prevention, while also paving the way for novel targeted therapeutic strategies in clinical practice. This review examines the core pathways of nucleotide metabolism, explores its relationship with PC, and highlights the significant alterations in nucleotide metabolism observed in PC.

## 2 Nucleotide metabolism

**Figure 1:**
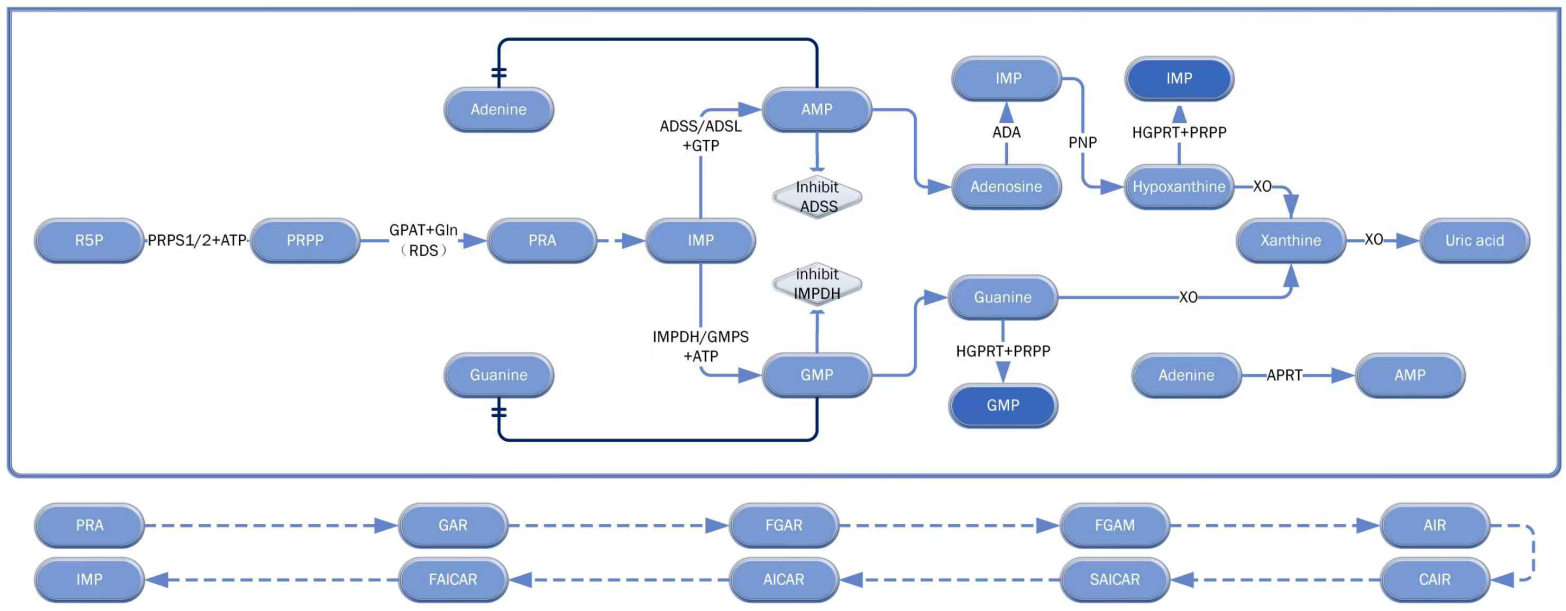
Core pathways of purine nucleotide metabolism: *de novo* synthesis, salvage synthesis, and catabolism. This diagram comprehensively illustrates the core biosynthetic and catabolic networks of purine nucleotides (e.g., AMP, GMP), covering the *de novo* synthesis, salvage synthesis, and catabolism of purines, along with some regulatory mechanisms. Solid arrows indicate the main metabolic pathways, while the dashed line indicates the process from PRA to IMP. The double-underlined arrows and diamond boxes highlight feedback inhibition, and the dark color emphasizes key components. This schematic diagram describes the various steps of purine metabolism as comprehensively as possible, and serves as a comprehensive reference for understanding purine nucleotide metabolism in normal pancreatic diseases. RDS, rate-determining step; R5P, Ribose-5-phosphate; PRA, Phosphoribosylamine.

**Figure 2:**
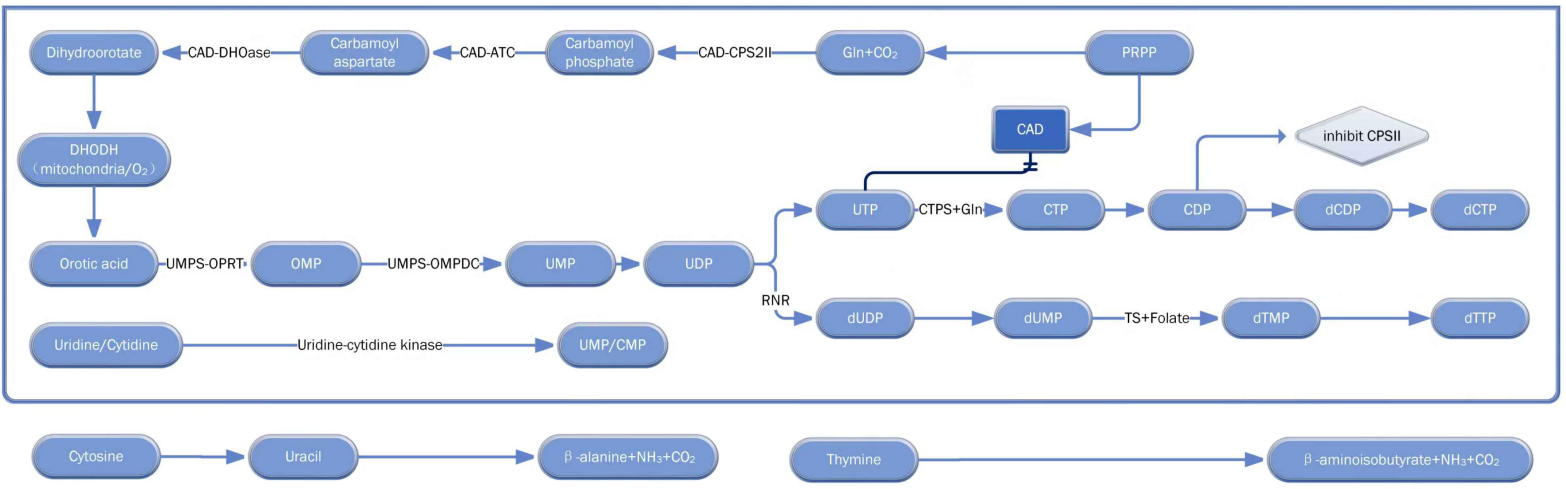
Core pathways of pyrimidine nucleotide metabolism: *de novo* synthesis, salvage synthesis, and catabolism. This diagram comprehensively illustrates the core biosynthetic and catabolic networks of pyrimidine nucleotides (e.g., UMP, CTP, dTTP), covering the *de novo* synthesis, salvage synthesis, and catabolism of pyrimidines, along with some regulatory mechanisms. The solid arrows represent the major metabolic flow. The double-underlined arrows and diamond boxes highlight feedback inhibition, and the dark color emphasizes key components. This schematic diagram describes the various steps of pyrimidine metabolism as comprehensively as possible, and serves as a comprehensive reference for understanding pyrimidine nucleotide metabolism in normal pancreatic diseases. OPRT, Orotate phosphoribosyltransferase; OMPDC, Orotidine-5’-monophosphate decarboxylase.

Nucleotide metabolism is a crucial metabolic pathway in the human body. Recent advancements, driven by global scientific collaboration, have progressively clarified the structure and function of key enzymes involved, shedding light on this complex and essential process. Nucleotide metabolism encompasses the *de novo* synthesis pathways of pyrimidines and purines, the salvage synthesis pathway (SSP), and nucleotide catabolism (**Figures 1**, **2**).

Typically, the *de novo* synthesis pathway is the primary route of nucleotide metabolism, while the SSP is utilized only in specific regions where relevant enzymes are insufficient. The *de novo* purine synthesis pathway begins with ribose-5-phosphate (R5P), which is enzymatically converted into 5-phosphoribose-1-pyrophosphate (PRPP) (9). PRPP is subsequently transformed into 5-phosphoribosamine (PAR), and through nine catalytic steps facilitated by five enzymes, PAR yields inosine monophosphate (IMP) (10). IMP is then converted to adenosine monophosphate (AMP) and guanosine monophosphate (GMP) by various enzymes (11). In purine salvage synthesis, hypoxanthine and guanine bind with PRPP to form IMP and GMP, respectively, while adenine is converted to AMP. The purine catabolic pathway involves the breakdown of AMP into hypoxanthine, which, along with guanine from GMP, is oxidized to xanthine and ultimately to uric acid by xanthine oxidase (XO). Excess AMP and GMP exert negative feedback inhibition on the production of adenylosuccinate synthetase (ADSS) and adenine, while excess GMP inhibits inosine monophosphate dehydrogenase (IMPDH) and guanine production.

The *de novo* pyrimidine synthesis pathway begins with the multifunctional CAD protein complex, which includes carbamoyl-phosphate synthetase II (CPSII), aspartate carbamoyltransferase (ATC), and dihydroorotase (DHOase) (12). This complex catalyzes the synthesis of carbamoyl phosphate, which, along with glutamine and aspartic acid, is ultimately converted into orotic acid through four key steps (13). As pyrimidine synthesis requires oxygen, it is closely linked to mitochondrial function and cellular oxygen availability. Orotic acid then undergoes reactions in the cytoplasm to form uridine monophosphate (UMP), which is subsequently converted into UDP and then into dCTP and dTTP. Under resting conditions or when pyrimidine demand is low, the body prefers the SSP. Uridine and cytidine are directly phosphorylated to UMP or CMP by intracellular enzymes, replenishing the pyrimidine pool through further reactions. Pyrimidine catabolism involves the degradation of cytosine and uracil into β-alanine, NH_3_, and CO_2_, while thymine is broken down into β-aminoisobutyric acid, NH_3_, and CO_2_.

## 3 Nucleotide metabolism and disease pathogenesis

### 3.1 Precancerous lesions

The pathogenesis of PC is a prolonged, multifactorial process, involving a series of genetic and cellular changes. It is characterized by various pathological alterations and progresses through several precursor lesions, including pancreatic acinar cell transformation (ADM), pancreatic intraepithelial neoplasia (PanIN), and intraductal pancreatic mucinous neoplasms (IPMN).

ADM refers to the reversible transdifferentiation of pancreatic acinar cells into duct-like cells. However, under the influence of carcinogenic drivers and chronic inflammation, ADM can progress to more advanced lesions, such as PanIN (14). PanIN lesions are typically small (< 5mm) and appear as flat or papillary structures within intralobular pancreatic ducts (15). They are classified based on nuclear atypia into three grades: PanIN-1 (low-grade), PanIN-2 (moderate-grade), and PanIN-3 (high-grade). IPMN, on the other hand, is marked by cystic lesions originating from the pancreatic ductal system, which produce mucin. These lesions are categorized into three subtypes: main duct IPMN (MD-IPMN), branch duct IPMN (BD-IPMN), and mixed-type IPMN (MT-IPMN) (16).

Notably, metabolic reprogramming occurs in these precursor lesions, indicating that early pancreatic carcinogenesis involves metabolic adaptations to meet the demands of nucleotide metabolism. Furthermore, metabolic alterations observed at the PanIN/IPMN stage appear to persist into the pancreatic ductal adenocarcinoma (PDAC) stage. In a metabolomic analysis of a PanIN mouse model, intermediates of the purine synthesis pathway did not increase as carcinogenesis progressed. In fact, levels of dAMP, GMP, and dGMP decreased. However, there was a noticeable increase in ADP and ATP levels, suggesting heightened cellular energy metabolism. This change may indicate either an enhancement or a subtle modification of the purine synthesis pathway, which warrants further investigation. In contrast, the pyrimidine synthesis pathway showed more pronounced upregulation during carcinogenesis. Levels of UDP and CMP increased, while the concentration of carbamoylaspartate, an early product of pyrimidine synthesis, decreased (17). In summary, current research indicates that alterations in these metabolites and their regulatory genes can indeed be observed in precancerous lesion models. However, whether these alterations act as functional drivers of disease occurrence remains unclear, and this gap highlights the need for further mechanistic studies.

### 3.2 Pancreatic carcinogenesis mechanisms

**Figure 3:**
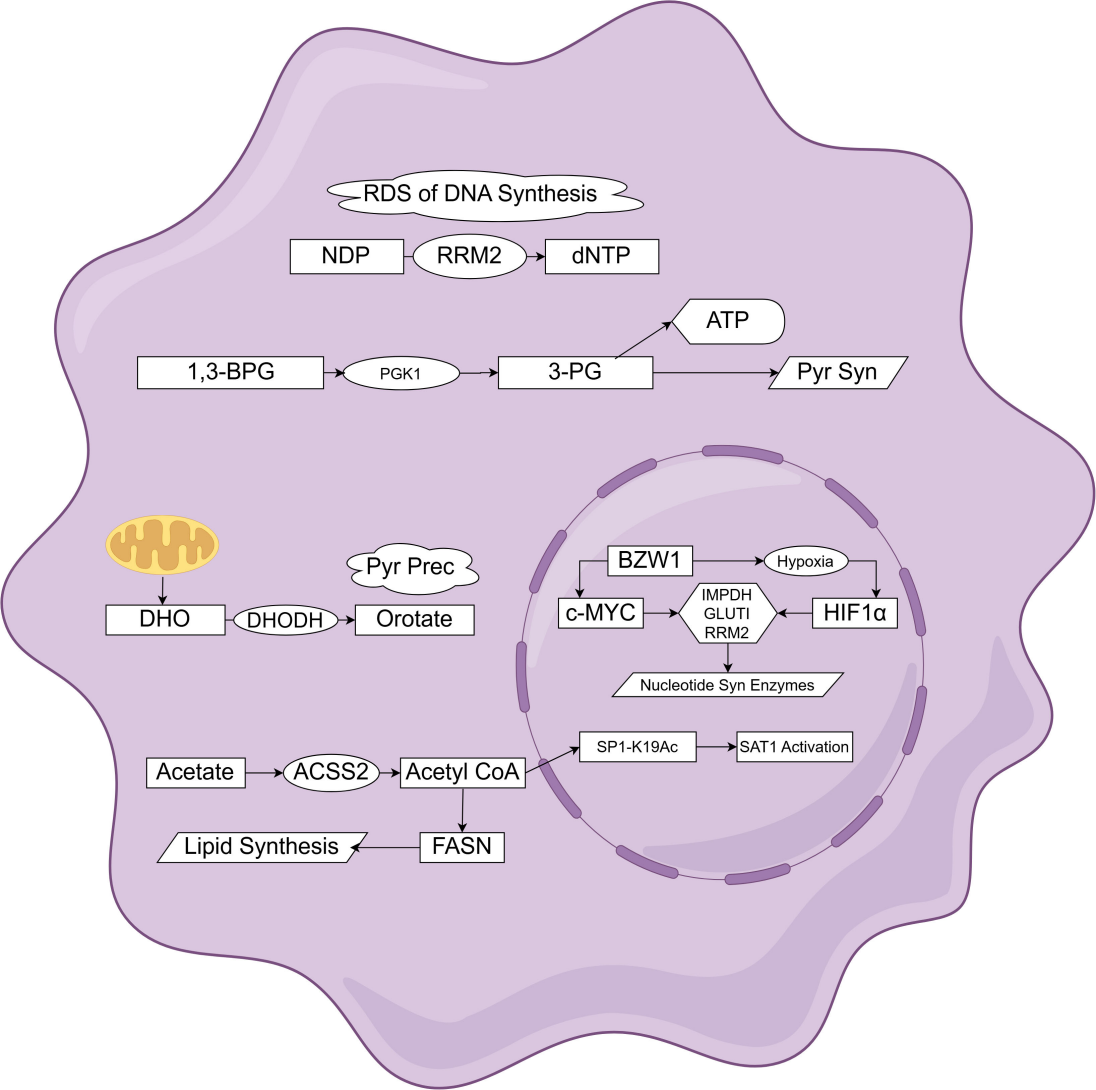
Schematic of nucleotide metabolism-related mechanisms in pancreatic cancer cells (by Figdraw). This diagram illustrates the mechanisms involved in nucleotide metabolism within pancreatic cancer cells, covering DNA synthesis, pyrimidine precursor production, nucleotide synthesis gene regulation, and acetate metabolism. Factors such as RRM2, PGK1, and DHODH, which are highly related to nucleotide metabolism in pancreatic cancer, play crucial functional and regulatory roles. This framework outlines the changes in nucleotide metabolic pathways in certain pancreatic cancer cells and highlights potential drug targets and regulatory mechanisms. RDS, rate-determining step; 1,3-BPG, 1,3-Bisphosphoglycerate; 3-PG, 3-Phosphoglycerate; Pyr Syn, Pyrimidine Synthesis; Pyr Prec, Pyrimidine Precursor; DHO, Dihydroorotate; SAT1 Act, SAT1 Activation.

PC is a highly lethal malignancy that can be classified into two major categories based on its cellular origin: pancreatic epithelial-derived and non-pancreatic epithelial-derived cancers. The majority of PC cases are pancreatic epithelial-derived, primarily comprising PDAC, which accounts for over 90% of cases. This category also includes adenosquamous carcinoma, colloid carcinoma, and undifferentiated carcinoma (18). Tumorigenesis in PDAC is driven by genomic instability, including somatic mutations, chromosomal rearrangements, copy number alterations, and epigenetic modifications. Two principal molecular models of PDAC pathogenesis provide differing views on tumor progression: one proposes a gradual, stepwise progression, while the other suggests a punctuated evolutionary pattern (19). Apart from genetic alterations, nucleotide metabolism-related mechanisms also play crucial roles in PDAC progression. Through a complex regulatory network, nucleotide metabolism-related mechanisms directly or indirectly affect PDAC progression. **Figure 3** shows the mechanism related to nucleotide metabolism in pancreatic cancer cells.

As in other solid tumors, angiogenesis plays a pivotal role in PC. It is considered a key rate-limiting step in both tumor growth and metastasis. In PC, angiogenesis is often induced by the hypoxic environment within the tumor. Tumor cells respond by producing and releasing a variety of growth factors, including vascular endothelial growth factor receptor (VEGFR) and neuropilin (NRP), which mediate and promote angiogenesis (20). Additionally, PC cells exhibit a unique pathological structure known as basal microvillus supply, which supports high metabolic activity. These structures are responsible for glucose transport into tumor cells and display endocytic properties similar to those of normal microvessels, facilitating nutrient exchange in the tumor microvasculature. This interaction may also enhance the activity of phagocytes and macrophages in PDAC (21).

The tumor microenvironment (TME) of PDAC consists primarily of various non-tumor cells, including cancer-associated fibroblasts (CAFs), endothelial cells (ECs), nerve cells, and immune cells, mainly myeloid cells. The TME is also rich in extracellular matrix (ECM) components such as growth factors, cytokines, hyaluronic acid (HA), and collagen. Notably, the TME of PDAC is strongly immunosuppressed, with a marked absence of highly active infiltrating CD8^+^ T cells (22). This unique immunosuppressive environment plays a critical role in promoting the proliferation, migration, and drug resistance of PDAC. PC cells meet the demands of rapid proliferation by upregulating purine and pyrimidine synthesis pathways. Additionally, the released metabolites can modulate the activity of immune cells (23, 24). Within the TME, tumor-associated macrophages (TAMs) undergo metabolic reprogramming from an M1 phenotype, which is pro-inflammatory, to an M2 phenotype, which is immunosuppressive. This shift inhibits the function of effector T cells, promoting tumor immune escape and progression. High concentrations of metabolites such as adenosine suppress T cell proliferation and cytotoxic function by binding to adenosine receptors on the surface of T cells (25, 26). Moreover, regulatory T cells (Tregs) accumulate in the PC microenvironment, further depleting critical metabolites like ATP and impairing the antitumor responses of effector T cells, thereby contributing to tumor immune evasion (27–29). Metabolic reprogramming within the TME alters the utilization of metabolites by immune cells, weakening their antitumor function and ultimately facilitating tumor growth and metastasis.

In addition to immune cells, current studies also implicate purinergic signaling in CAF and PDAC crosstalk. In PDAC the CD39/CD73 pathway (ATP-AMP-adenosine axis) is markedly upregulated, yielding high extracellular adenosine that suppresses CD8+ T and NK cells while promoting Tregs and MDSCs (30). Importantly, CAFs and tumor cells are major sources of this pathway (30, 31). CAFs express CD73 (NT5E) and other ectonucleotidases to produce adenosine, reinforcing local immune evasion (30, 31). For example, multiscale profiling in PDAC found that CD73+ CAFs cluster near tumor cells and likely mediate metabolic crosstalk and immunosuppression in the dense stroma (31). Single-cell analysis and spatial data show that PDAC cells and CAFs are accompanied by higher scores of purine metabolism (32). Co-culture experiments further show that silencing NT5E (CD73) in PDAC cells reduced their invasion/proliferation, but this effect was largely rescued by co-culture with CAFs (32). These findings collectively indicate that CAF‐derived purine metabolites and enzymes drive PDAC progression and immune suppression via metabolic reprogramming of the tumor microenvironment (e.g. ATP/AMP conversion to adenosine). Targeting this CAF-purine axis may represent a strategy to disrupt tumor‐promoting metabolic symbiosis and restore anti‐tumor immunity in PDAC.

### 3.3 Changes in nucleotide metabolism-related genes and enzymes in pancreatic diseases

The role of nucleotide metabolism in pancreatic diseases has garnered increasing attention, with numerous related genes and their functions now being identified. **Table 1** summarizes several relevant genes, while **Table 2** (33–81) provides a brief overview of the mechanisms associated with these genes. Below are some key changes in genes and enzymes.

**Table 1:** Forty-two genes associated with nucleotide metabolism.

ADA	ADK	ADSL	AK1	AK2	AK4
CAD	DCK	DGUOK	DHODH	DPYD	DPYS
DTYMK	DUT	GART	GDA	GLRX	GMPR
GSR	ITPA	KRAS	LHPP	NT5C	NT5C2
NT5E	NT5DC	NUDT1	NUDT15	NUDT16	NUDT18
PAICS	RRM1	RRM2	RRM2B	SAMHD1	TK1
TK2	TXNRD1	TYMP	TYMS	UMPS	XDH

**Table 2:** Genetic mechanisms of the 42 genes: brief elaboration.

Target gene name	Relevant mechanism	References
ADA	Mediates the deamination of adenosine and deoxyadenosine to generate inosine and deoxyinosine, critical for purine metabolism homeostasis	(33)
ADK	Regulates intracellular adenosine levels through phosphorylation catalysis, modulating neuronal transmission and energy metabolism	(34–36)
ADSL	Catalyzes the cleavage of adenylosuccinate and succinyl adenosine in the purine biosynthesis pathway, yielding fumarate and AMP	(37, 38)
AK1	Maintains adenylate equilibrium through ATP+AMP ↔ 2ADP interconversion, crucial for cellular energy metabolism	(39)
AK2	Overexpression activates the TGF-β/Smad3/Smad2/Smad4 axis, promoting EMT-mediated tumor invasiveness	(40)
AK4	Modulates mitochondrial ATP/AMP flux, coordinating cellular energy balance and stress responses	(41)
AMPD1	Converts AMP to IMP in muscle; deficiency leads to AMP accumulation, causing impaired energy metabolism manifesting as post-exertional myalgia and fatigue	(42)
CAD	Multifunctional enzyme initiating pyrimidine biosynthesis: carbamoyl phosphate synthesis→carbamoyl aspartate formation→dihydroorotate production	(43)
DCK	Initiates phosphorylation of deoxyribonucleosides (dCyd, dGuo, dAdo) in the nucleoside salvage pathway	(44–46)
DGUOK	Phosphorylates deoxyguanosine to dGMP; mutations disrupt mitochondrial DNA replication	(47)
DPYD	Encodes dihydropyrimidine dehydrogenase (DPD) that catalyzes pyrimidine catabolism to uracil/thymine, determining 5-FU pharmacokinetics	(48)
DPYS	Mediates reversible hydrolytic ring-opening of dihydropyrimidines: 5,6-dihydrouracil→N-carbamoyl-β-alanine; 5,6-dihydrothymine→N-carbamoyl-α-aminoisobutyrate	(49)
DTYMK	Phosphorylates dTMP to dTDP in pyrimidine metabolism, essential for DNA replication fidelity	(50)
DUT	Hydrolyzes dUTP to dUMP, preventing dUTP misincorporation into DNA strands (critical for genomic stability)	(51)
GART	Trifunctional enzyme in *de novo* purine synthesis: phosphoribosylglycinamide formyltransferase (GAR Tfase)/synthetase (GARS)/AIR synthetase (AIRS) activities	(52)
GDA	Catalyzes guanine to xanthine conversion in the purine degradation pathway, maintaining epidermal homeostasis	(53)
GLRX	Glutaredoxin system component regulating redox homeostasis through protein disulfide reduction	(54)
GMPR	Converts GMP to IMP *via* NADPH-dependent deamination, balancing purine nucleotide pools	(55)
GSR	Reduces oxidized glutathione (GSSG) to GSH using NADPH (EC 1.8.1.7), crucial for redox homeostasis	(56)
ITPA	Hydrolyzes non-canonical nucleotides: ITP→IMP, dITP→dIMP, XTP→XMP (EC 3.6.1.19)	(57, 58)
KRAS	The protein encodes a member of the small GTPase superfamily. A single amino acid substitution results in an activating mutation.	(59)
LHPP	Histidine/lysine phosphatase (EC 3.6.1.3) regulating PI3K/AKT/mTOR signaling network	(60)
NT5C	5’(3’)-nucleotidase (EC 3.1.3.5) dephosphorylating deoxyribonucleotides, regulating dNTP pools	(61)
NT5C2	Gain-of-function mutations enhance chemoresistance to mercaptopurine *via* CMP hydrolysis-mediated reduction of active drug metabolites	(62)
NT5DC	The coding sequence contains the 5’-nucleotidase domain (NT5DC) family.	(63)
NT5E	Encodes CD73 (ecto-5’-nucleotidase, EC 3.1.3.5) catalyzing ATP/ADP→adenosine conversion, suppressing anti-tumor immunity	(64)
NUDT1	Sanitizes oxidized nucleotides (8-oxo-dGTP→8-oxo-dGMP, EC 3.6.1.12) preventing DNA mutagenesis	(65)
NUDT15	Cleaves thio-dGTP/dTTP/dCTP (EC 3.6.1.1), determining thiopurine drug metabolism efficiency	(66)
NUDT16	Prevents mutagenic nucleotide incorporation *via* IMP/XMP hydrolysis (EC 3.6.1.1)	(67)
NUDT18	Hydrolyzes 8-oxo-dGTP (EC 3.6.1.12) in the nucleotide pool sanitation pathway	(68)
PAICS	Catalyzes AICAR→SAICAR conversion (EC 6.3.2.6) in *de novo* purine biosynthesis	(69)
RRM1	Catalytic subunit of ribonucleotide reductase (EC 1.17.4.1), converts NDP→dNDP with allosteric regulation	(70)
RRM2	Radical-generating subunit of ribonucleotide reductase, requires iron cofactor for catalysis	(71)
RRM2B	p53-inducible isoform (EC 1.17.4.1) maintaining dNTP pool balance during DNA repair	(72)
SAMHD1	dNTP triphosphohydrolase (EC 3.1.5.1) restricting retroviral replication *via* dNTP depletion	(73)
TK1	Cell cycle-regulated thymidine kinase (EC 2.7.1.21), biomarker for tumor proliferation	(74)
TK2	Mitochondrial deoxyribonucleoside kinase (EC 2.7.1.113) essential for mtDNA maintenance	(75)
TXNRD1	Thioredoxin reductase (EC 1.8.1.9) maintaining thioredoxin in reduced state using NADPH	(76)
TYMP	Thymidine phosphorylase (EC 2.4.2.4) generating 2-deoxy-D-ribose-1-phosphate for neovascularization	(77)
TYMS	Thymidylate synthase (EC 2.1.1.45) mediating dUMP→dTMP conversion with 5,10-CH2-THF cofactor	(78, 79)
UMPS	Bifunctional enzyme (EC 2.4.2.10 & 4.1.1.23) converting orotate→UMP *via* OMP intermediate	(80)
XDH	Xanthine oxidoreductase (EC 1.17.3.2) producing uric acid *via* hypoxanthine→xanthine oxidation	(81)

#### 3.3.1 Purine metabolism related

The elevated expression of adenosine succinate lyase (ADSL) in cancer is linked to tumor invasion and poor prognosis. This effect is mediated through the inhibition of Carma3 expression, which influences resistance to gemcitabine (also known as 2,2-difluorodeoxycytidine, dFdC), while Nrf2 signaling can regulate ADSL expression. Knockdown of ADSL significantly reduces the responsiveness of PC cells to gemcitabine treatment (82).

Adenosine deaminase (ADA) plays a role in adenosine metabolism by converting adenosine to hypoxanthine. Although its precise role in PC remains unclear, it is known that the serum levels of ADA in patients with pancreatic diseases differ significantly from those in healthy individuals (83), particularly in patients with PC, making it a potential area for further investigation. CD73 (NT5E), a key enzyme that converts AMP to adenosine, has been explored in PC, with the CD73 inhibitor AB680 showing promise (84). Several CD73 inhibitors are currently in clinical trials (85). Research on the Nudix hydrolase superfamily in PC is still limited. These enzymes hydrolyze toxic nucleoside triphosphates, and NUDT15 has emerged as a potential biomarker (86), with its high expression strongly correlating with early postoperative recurrence risk. Further investigation into its underlying mechanisms is needed.

Adenylate kinase (AK) regulates multiple cellular functions, including maintaining adenine nucleotide metabolic homeostasis, activating the AK-AMP-AMPK signaling pathway, regulating the cell cycle, proliferation, and intracellular energy transfer, as well as mitochondrial ATP distribution. Studies suggest that AK expression is upregulated in metastatic pancreatic endocrine tumors, with overexpression potentially promoting tumorigenesis. AK also influences the efficacy of adjuvant therapy by inducing epithelial-mesenchymal transition (EMT). Compared to normal tissues, AK2 expression is elevated in PDAC, though its exact role requires further exploration (87). In studies of adenylate kinase 4 pseudogene 1 (AK4P1), both AK4 and AK4P1 were identified as oncogenic and significantly upregulated in PDAC (88).

Phosphoribosylaminoimidazole succinocarboxamide synthetase (PAICS), which catalyzes the conversion of SAICAR to AICAR in purine biosynthesis, is overexpressed in PDAC. Research indicates that its knockdown suppresses cell proliferation, colony formation, invasion, motility, and spheroid formation, suggesting that PAICS targeting may offer a promising therapeutic strategy for PDAC (69).

The NT5DC family includes evolutionarily conserved 5’-nucleotidases that catalyze intracellular nucleotide hydrolysis. Recent studies suggest that NT5DC2 may serve as both a therapeutic target and a valuable biomarker for personalized treatment in patients with PC (89).

#### 3.3.2 Pyrimidine metabolism related

Upregulation of dihydropyrimidine dehydrogenase (DPYD), which catalyzes the catabolism and inactivation of 5-fluorouracil (5-FU) in pyrimidine-based chemotherapy, has been linked to increased proliferation, invasion, angiogenesis, and resistance to 5-FU treatment in PC. Elevated DPYD expression in PDAC not only enhances pyrimidine degradation but also promotes cell proliferation and invasiveness, accompanied by upregulation of MMP9 and MEP1A (90). This suggests potential therapeutic benefits of targeting DPYD in clinical settings.

Dihydroorotate dehydrogenase (DHODH), a key enzyme in *de novo* pyrimidine nucleotide synthesis, has demonstrated promising preclinical activity. However, DHODH inhibitors have largely failed to show efficacy in PDAC and other solid tumors in multiple clinical trials, with cancer cells seemingly evading inhibition of this metabolic enzyme. The underlying mechanisms remain unclear, and further investigations are ongoing (91, 92). Teriflunomide, the active metabolite of the immunosuppressant leflunomide, directly inhibits DHODH and has been used in the treatment of rheumatoid arthritis (93). Its potential role in PC remains to be explored.

Research on the TYMS gene in PC is gradually progressing. Literature suggests that TYMS is upregulated in PC, with varying expression levels across different histological grades and clinical stages. High TYMS expression is associated with poor prognosis in patients (94). Additionally, the TYMS gene encodes thymidylate synthase (TS).

TS plays a pivotal role in the synthesis of deoxythymidine monophosphate (dTMP) by catalyzing the conversion of deoxyuridine monophosphate (dUMP) to dTMP. As one of the earliest identified anti-cancer targets (95), its role in PC remains incompletely understood. Some studies report that TS activity in PC is significantly higher than in normal pancreatic tissue, though lower than in other solid tumors (96). Additionally, high TS expression correlates with advanced clinical stages and poor prognosis, making TS a potential biomarker for the diagnosis and prognosis of patients with PC (94).

5-FU, widely used in the treatment of various gastrointestinal cancers, including PC, targets TS. Although 5-FU’s efficacy in PC tissue may be lower than in normal tissue (96), it remains a cornerstone of treatment. As a TS inhibitor, 5-FU interferes with dTMP production, thereby inhibiting DNA synthesis in cancer cells (97, 98). Standard regimens like FOLFIRINOX (oxaliplatin, irinotecan, 5-FU, and leucovorin) leverage the effects of 5-FU and are commonly used for the initial treatment of metastatic pancreatic adenocarcinoma (MPC) (99, 100). Overexpression of TS has been closely linked to 5-FU resistance. 5-FU binds to TS through its active metabolite, fluorodeoxyuridine monophosphate (FdUMP), inhibiting TS activity and disrupting DNA synthesis. However, TS overexpression diminishes the therapeutic effects of 5-FU. Therefore, inhibiting TS activity can enhance 5-FU efficacy (101, 102). Moreover, combining strategies to synergistically inhibit TS activity may further improve therapeutic outcomes. Tumor genotypes and metabolic adaptations in the TME also modulate TS activity, highlighting the need for personalized TS-targeted therapies based on patient stratification (103, 104).

Deoxycytidine kinase (DCK) is a key enzyme involved in the SSP of deoxyribonucleotides and is crucial for the phosphorylation of cytidine, thus playing an essential role in maintaining normal DNA metabolism. Given that DCK affects the metabolism of gemcitabine, a first-line nucleoside analog drug used to treat PC, much of the existing literature focuses on its role in gemcitabine metabolism, though its direct impact on PC remains underexplored. Some studies indicate that in idiopathic pulmonary fibrosis (IPF), DCK is a downstream target of hypoxia and contributes to alveolar epithelial cell proliferation, while in chronic obstructive pulmonary disease (COPD), elevated DCK levels can trigger apoptosis in chronic lung disease cells (105). However, it is clear that during PC treatment with gemcitabine, DCK expression decreases as the disease progresses (106). Research into DCK’s regulatory mechanisms in PC is still incomplete and warrants further investigation. Clinically, gemcitabine is widely used as a standard treatment for advanced PC. Decreased expression or mutations in DCK are closely associated with gemcitabine resistance, particularly in PC. As a key enzyme in gemcitabine activation, reduced DCK activity results in lower cellular uptake and activation of the drug, thereby compromising its efficacy (107). Increasing DCK expression or activity can enhance the cytotoxic effects of gemcitabine. Specifically, certain metabolic inhibitors may reactivate DCK by modifying the tumor’s metabolic environment, thus restoring gemcitabine efficacy (108). Furthermore, some studies have explored gene therapy approaches to directly introduce the DCK gene into PC tumors. This strategy, when combined with chemotherapy, not only increases DCK activity but also amplifies the drug’s cytotoxicity against cancer cells.

Ribonucleotide reductase M2 (RRM2), a subunit of ribonucleotide reductase (RNR), is responsible for converting ribonucleotides to deoxyribonucleotides, a key step in DNA synthesis. Literature suggests that high RRM2 expression in PC is associated with poor survival rates. Silencing RRM2 inhibits PC cell proliferation and tumor growth by inactivating the PI3K/AKT/mTOR pathway, leading to cell cycle arrest and/or apoptosis (109). As an RNR-inhibiting antimetabolite, gemcitabine remains one of the few FDA-approved drugs for PC treatment (110). Currently, more selective RRM2 inhibitors are under development.

#### 3.3.3 Oncogenic driver genes and other critical genes

The Kirsten Rat Sarcoma (KRAS) gene mutation is the most prevalent mutation across all cancers, including PC. Over 90% of PDAC cases harbor activated KRAS mutations, which are strongly associated with disease progression (111). The KRAS gene encodes a small GTPase protein that functions as a molecular switch for numerous key intracellular signaling pathways. KRAS activity is determined by its binding to either GTP, which activates it, or GDP, which inactivates it. The most common KRAS mutations occur at codon 12 of the oncogene, and include G12D, G12V, and G12R. Oncogenic KRAS activates several critical downstream effector pathways, including the RAF-MEK-ERK MAPK pathway, the PI3K-AKT-mTOR pathway, and the Ral guanine nucleotide exchange factor (RalGEF) pathway. Direct pharmacological targeting of KRAS has historically been considered challenging, but recent studies have identified viable therapeutic strategies for KRAS-targeted therapy (112, 113). Moreover, nucleotide metabolism is a key mediator of KRAS resistance, with oncogenic KRAS contributing to PC progression by regulating nucleotide metabolism (114).

The oncogenic KRAS gene plays a pivotal role in pancreatic disease progression through its interactions with nucleotide metabolism. While past research has primarily targeted its downstream signaling pathways, significant clinical advancements remain elusive (115–117). The link between KRAS and nucleotide metabolism still requires further exploration. Sotorasib and Adagrasib, two KRAS G12C inhibitors, have shown efficacy in various cancers, including non-small cell lung cancer (NSCLC) (118, 119). However, their clinical efficacy in KRAS-mutant PCs is limited by drug resistance and transient therapeutic effects. Although these inhibitors demonstrate potent tumor-suppressive activity during initial treatment (112), acquired resistance inevitably develops with prolonged therapy. These resistance mechanisms extend beyond KRAS itself to include the activation of downstream signaling pathways such as the MAPK and PI3K-AKT-mTOR pathways (120–122). KRAS-mutant tumor cells often rely on enhanced nucleotide metabolic pathways to support rapid cell proliferation. KRAS G12C inhibitors can inhibit tumor growth by altering the metabolic state of tumor cells, particularly affecting the nucleotide synthesis pathway (123). Although KRAS G12C inhibitors show some efficacy when used alone (124), literature suggests that combination therapies may offer a promising direction for overcoming resistance and improving tumor suppression. Clinical exploration of such combination therapies is ongoing (121, 125).

The phospholysine phosphohistidine inorganic pyrophosphate phosphatase (LHPP) catalyzes the removal of phosphohistidine and phospholysine modifications from target proteins. LHPP is significantly downregulated in PC tissues and cell lines, and its expression suppresses PC cell proliferation, migration, and invasion while promoting apoptosis through AKT signaling (126, 127). LHPP holds promise not only as a therapeutic target but also as a prognostic biomarker and metabolic regulator, offering novel insights for PC management.

### 3.4 Advances in targeting nucleotide metabolism for PC therapy and related clinical trials

KRAS has emerged as a central focus of research in PDAC (128). Recent advances have been made in developing direct inhibitors targeting the KRAS-G12C mutation, which have shown promise in treating certain solid tumors. The Phase I/II CodeBreaK 100 trial demonstrated positive effects in PDAC treatment (129), though the efficacy of KRAS inhibitor monotherapy remains limited. Ongoing investigations are exploring adagrasib for KRAS-G12C-mutated PC, with clinical studies examining combination strategies involving KRAS inhibitors and chemotherapy. Furthermore, combining KRAS inhibitors with immunotherapy holds significant potential. Preclinical studies suggest that KRAS-G12C inhibitors may enhance tumor immunogenicity, potentially synergizing with immunotherapies. Building on these findings, the CodeBreaK 101 trial is evaluating sotorasib in combination with pembrolizumab or atezolizumab.

Small molecule inhibitors targeting PAICS are currently under development. Computer-screened PAICS inhibitory compounds have demonstrated some inhibitory effects on PDAC cells (130). Although no clinical trial results are available yet, further in-depth studies are warranted. TS has long been a target of chemotherapy. 5-FU, its derivatives, and gemcitabine are commonly used chemotherapeutic agents in various combination regimens. Efforts are underway in the pharmaceutical field to develop drugs that bypass DCK to overcome DCK-induced gemcitabine resistance (131). Research on ADSL, LHPP, DPYD, RRM2, ADA, and the NT5DC family is ongoing, though clinical trials remain limited. Future advancements are eagerly anticipated.

Moreover, multiple combination therapeutic strategies are being explored, including KRAS inhibitors combined with metabolic pathway inhibition, direct metabolic inhibition paired with immunotherapy, multi-metabolic pathway suppression integrated with targeted therapy, and metabolic reprogramming interventions alongside immunotherapy. However, current research faces several challenges, including suboptimal efficacy, significant population heterogeneity, incompletely controlled overlapping drug toxicities, and excessive physiological burdens on patients. The underlying resistance mechanisms—whether known or yet to be fully characterized—require further investigation.

## 4 Conclusion and outlook

As insights into the role of nucleotide metabolism in PC continue to deepen, clinical diagnostic and drug development targets are gradually becoming clearer. In terms of early screening and diagnosis, KRAS is the only gene currently with clinically translatable potential for early detection. While PAICS and RRM2 show upregulation at the histological level, their use as early diagnostic biomarkers remains distant. NUDT15, DPYD, TYMS, and DCK hold promise as prognostic markers, while ADSL, ADA, AK, NT5DC, and LHPP exhibit limited potential based on current evidence.

Although the impact of nucleotide metabolic pathways on tumorigenesis and progression is increasingly recognized, and their medical significance shows promise, numerous critical questions remain unresolved. These include the relationship between nucleotide metabolism and early diagnostic biomarkers, the synergistic effects of immunotherapy combined with nucleotide metabolism inhibitors, the cross-tissue and microenvironmental effects of nucleotide metabolism, and the development and clinical application of small molecule inhibitors.

In conclusion, the study of nucleotide metabolism in pancreatic diseases has significant scientific implications and potential clinical applications. Future research is expected to bring breakthroughs in this field, offering new strategies and directions for the treatment of pancreatic diseases.
